# Stress-related disorders and subsequent cancer risk and mortality: a population-based and sibling-controlled cohort study in Sweden

**DOI:** 10.1007/s10654-022-00898-x

**Published:** 2022-08-13

**Authors:** Fan Tian, Fang Fang, Qing Shen, Weimin Ye, Unnur A. Valdimarsdóttir, Huan Song

**Affiliations:** 1 West China Biomedical Big Data Center, West China Hospital, Sichuan University, Guo Xue Lane 37, 610041 Chengdu, China; 2 West China School of Public Health and West China Fourth Hospital, Sichuan University, Chengdu, China; 3 Institute of Environmental Medicine, Karolinska Institute, Stockholm, Sweden; 4 Department of Medical Epidemiology and Biostatistics, Karolinska Institute, Stockholm, Sweden; 5 Center of Public Health Sciences, Faculty of Medicine, University of Iceland, Reykjavík, Iceland; 6 Department of Epidemiology, Harvard T.H. Chan School of Public Health, Boston, USA; 7 Med-X Center for Informatics, Sichuan University, Chengdu, China

**Keywords:** Stress-related disorders, Post-traumatic stress disorder, Reaction to severe stress, Malignant neoplasms, Cancer

## Abstract

Prior research has suggested a potential role of psychological stress on cancer development while the role of familial factors on this association is underexplored. We conducted a nationwide cohort study including 167,836 individuals with a first-onset stress-related disorder (including post-traumatic stress disorder, acute stress reaction, adjustment disorder and other stress reactions) diagnosed between 1981 and 2016 in Sweden (i.e., exposed patients), 1,631,801 birth year- and sex-matched unexposed individuals, and 179,209 unaffected full siblings of the exposed patients. Cox models were used to estimate the hazard ratios (HRs) of newly diagnosed cancer and cancer-related death, beyond 1 year after diagnosis of stress-related disorders. We further examined the potential mediation roles of behavior-related morbidities in the associations of stress-related disorders with smoking or alcohol-related cancer incidence and mortality. We found modestly elevated risks of cancer incidence and mortality among exposed patients compared with matched unexposed individuals (incidence: HR = 1.03, 95% CI 1.01–1.06; mortality: HR = 1.13, 95% CI 1.07–1.18), while not when comparing with full siblings (incidence: HR = 1.03, 95% CI 0.99–1.08; mortality: HR = 1.09, 95% CI 1.00-1.19). Similarly, the suggested elevations in incidence and mortality of individual cancer sites (or groups) in the population-based comparison attenuated towards null in the between-sibling comparison. The risk elevations for smoking or alcohol-related cancers in the population-based comparison (incidence: HR = 1.18, 95% CI 1.11–1.24; mortality: HR = 1.20, 95% CI 1.12–1.29) were partially mediated by alcohol-related morbidities during follow-up. Collectively, our findings suggest that the association between stress-related disorders and cancer risk and mortality is largely explained by familial factors, including shared behavioral hazards.

## Introduction

The potential role of psychological stress on cancer initiation and progression has received comprehensive scientific efforts during the past decades. Accumulating evidence with a focus on experiences of stress [1–4] (e.g., loss of a child or parent due to death, divorce, daily stress, and care-giver stress) and psychiatric disorders (e.g., depression [5, 6] and anxiety [6]) suggests an increase in the incidence of some cancers and cancer-related mortality among individuals exposed to these stress-related conditions. Proposed underlying mechanisms include impaired endocrine and immune function [7, 8], accumulation of somatic mutations [9], inhibited repair of damaged DNA [10], and stress-induced adverse behaviors [1, 11] (e.g., smoking, alcohol abuse, unfavorable diet, and physical inactivity).

Stress-related disorders are a cluster of psychiatric disorders, including acute stress reaction, post-traumatic stress disorder (PTSD), adjustment disorder and other stress reactions, resulting from a preceding traumatic or stressful life event [12]. Patients with stress-related disorders often present with physiological dysfunction as a consequence of severe stress reaction [8]. Stress-related disorders have also been linked to subsequently elevated risks of multiple somatic diseases, such as cardiovascular disease [13], life-threatening infections [14], and neurodegenerative diseases [15]. However, evidence on the potential role of stress-related disorders on cancer incidence and mortality remains limited.

A prospective cohort study of 116,429 female nurses indicated a positive association between PTSD symptoms and risk of ovarian cancer incidence [16]. Another study of 15,288 Vietnam veterans with up to 30 years of follow-up suggested an increased cancer-specific mortality among veterans with PTSD [17]. A register-based study in Denmark of 4,131 individuals with PTSD found however no association between PTSD and overall cancer incidence [18]. Null findings were also subsequently reported for adjustment disorder and any type of cancer, except for smoking or alcohol-related cancers [19]. A previous study based on the UK population, did not find any association between PTSD and risk of lung, breast, prostate, and colorectal cancers [20]. The inconsistency of the existing evidence may be explained by the different stress-related disorders and different types of cancers studied [16, 20], as well as methodological heterogeneity, e.g., different definitions of PTSD [16, 18], varying study sample sizes [16, 20], and different degree of control for important confounding factors [16, 20]. A comprehensive assessment of the impact of all stress-related disorders on the development of different cancer types is therefore needed. Also, cancer development is partly attributed to familial (genetic and shared environmental) factors [21], which has not been addressed in most existing studies. To this end, we leveraged the nationwide population registers in Sweden with sibling-controlled design, to examine the associations between stress-related disorders and subsequent risk of cancer incidence and mortality while controlling for familial confounding, socioeconomic status, and history of various somatic diseases.

## Methods

### Study design

**Fig. 1:**
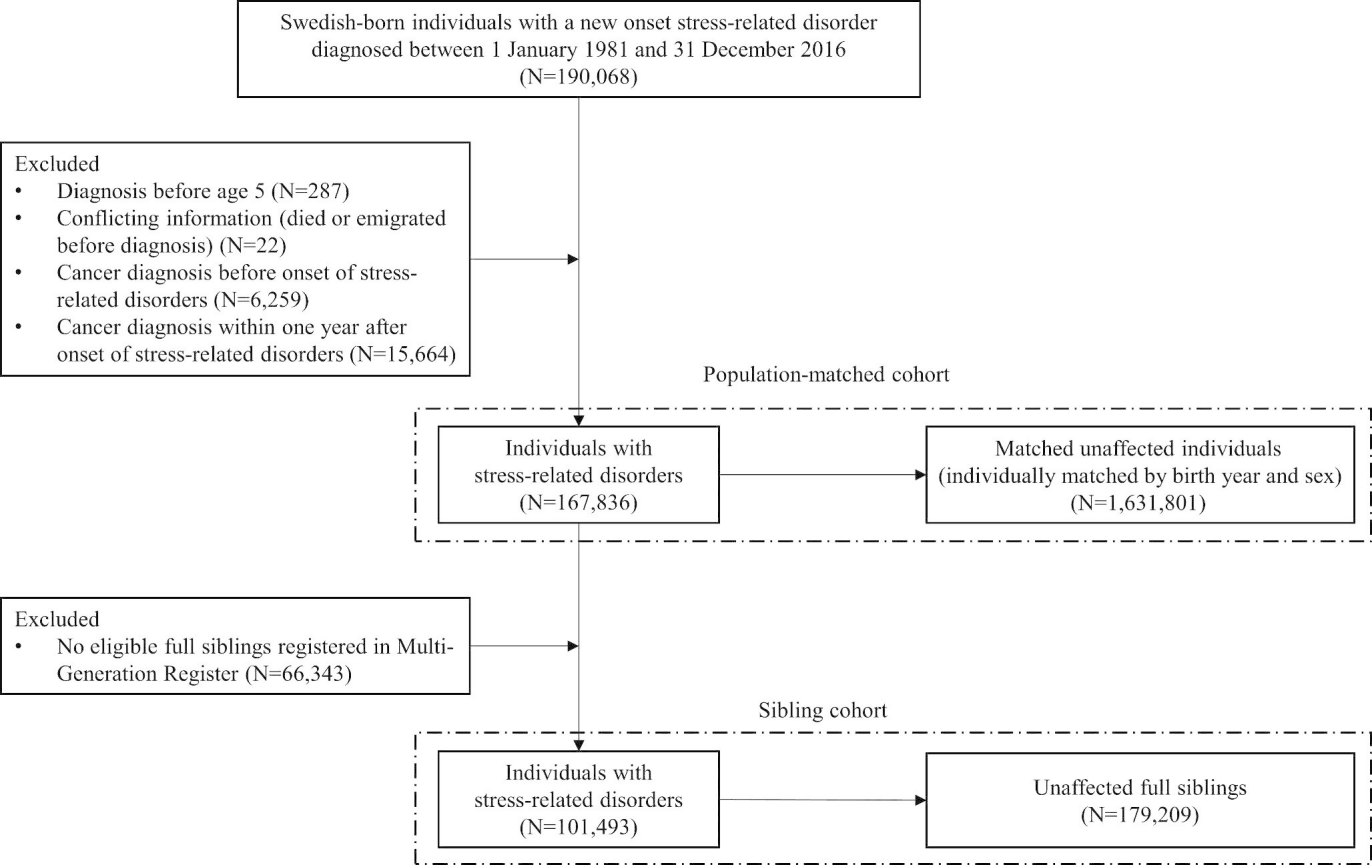
Study design

Based on the Swedish Total Population Register [22], we identified 8,753,501 individuals born in Sweden between 1932 and 2011. Using the national registration numbers uniquely assigned to all Swedish residents, we linked the data to the Swedish National Patient Register [23] and identified all individuals who received their first diagnosis of stress-related disorders between 1 January 1981 and 31 December 2016 as the exposed group (N = 190,068; Fig. 1). We obtained information for the exposed individuals through cross-linkage to the Swedish Cancer Register [24], Causes of Death Register [25], and Migration Register. We excluded those with a diagnosis of stress-related disorders at age 5 or younger [26] (N = 287), conflicting information (died or emigrated before diagnosis) (N = 22), or a history of cancer diagnosis before the onset of stress-related disorders (N = 6,259). To alleviate the concern of reverse causality (i.e., stress-related disorders might be subsequent to pre-clinical cancer symptoms [27]), we further excluded those with a cancer diagnosis within one year after the onset of stress-related disorders (N = 15,664), leaving 167,836 eligible exposed individuals in the analysis.

We constructed a population-based matched cohort based on the Swedish Total Population Register [22] using the method of incidence density sampling [28]. For each exposed person (i.e., the index patient), up to 10 individuals who were free of stress-related disorders and cancer at the diagnosis date of the index patient (i.e., the index date for both exposed and unexposed individuals) were randomly selected and individually matched to the index patient by birth year and sex. We included 1,631,801 unexposed individuals in the analysis.

To control for familial confounding due to shared genetic background and environmental factors, we further constructed a between-sibling comparison to compare the exposed individual with their unaffected full siblings. Based on the Swedish Multi-Generation Register [29], we identified all clusters of full siblings that were discordant for stress-related disorders, including 179,209 unaffected full siblings of 101,493 exposed individuals (Fig. 1). The unaffected full siblings were free of stress-related disorders and cancer at the diagnosis date of the affected sibling (i.e., the index date for both affected and unaffected siblings).

## Follow-up

Both cancer incidence and cancer mortality were outcomes of interest in this study, we therefore defined two endpoints for the follow-up. Because stress-related disorders might be subsequent to pre-clinical cancer symptoms, we started the follow-up of all study participants from 1 year after the index date. For cancer incidence, follow-up started from 1 year after the index date to the date of first cancer diagnosis, death, emigration, or the end of follow-up (December 31, 2016), whichever occurred first. For cancer mortality, follow-up was from 1 year after the index date to the date of death, emigration, or the end of follow-up (December 31, 2016), whichever came first. The follow-up for matched unexposed individuals or unaffected full siblings was additionally censored at the diagnosis of stress-related disorders, if any, during the follow-up. These patients were then included in the exposed cohort.

## Ascertainment of stress-related disorders

We defined stress-related disorders as any first inpatient or outpatient visit with a primary diagnosis of stress-related disorders in the National Patient Register, according to the 8th, 9th, or 10th Swedish revisions of the International Classification of Diseases (ICD) codes 307 (ICD-8), 308 or 309 (ICD-9), and F43 (ICD-10) (Supplementary Table 1). The subtypes of stress-related disorders were identifiable in 1987 and onward when ICD-9 was used in the National Patient Register, we therefore further divided individuals with a diagnosis of stress-related disorders from 1987 onward as PTSD (ICD-9: 309B; ICD-10: F43.1), acute stress reaction (ICD-9: 308, 309 A; ICD-10: F43.0), and adjustment disorder and other stress reactions (ICD-9: 309X, 309B; ICD-10: F43.2, F43.8, F43.9). Considering other stress-related disorders (e.g., acute stress reaction [30]) might be a precursor to the development of subsequent PTSD, all exposed individuals with PTSD in the analysis were defined as those who received a PTSD diagnosis within one year after their first stress-related disorder diagnosis.

## Cancer incidence and mortality

Cases with incident cancer were ascertained based on diagnosis in the Swedish Cancer Register, according to ICD-7 codes 140–209. This register contains nationwide information on all newly diagnosed cases of cancer in Sweden since 1958 [24]. Deaths due to cancer were identified by the underlying cause of death documented in the Causes of Death Register. In sub-analysis, we classified the malignances according to sites as follows: esophageal cancer, gastric cancer, colorectal cancer, liver cancer, pancreatic cancer, lung cancer, skin cancer, breast cancer, head and neck cancer, prostate cancer, central nervous system (CNS) cancer, lymphatic or hematopoietic malignancies, and other cancers. We further divided cancers into four major categories according to the similarities in etiologies [31], including hematological malignancies, hormone-related cancers, immune-related cancers, and smoking or alcohol-related cancers (Supplementary Table 1).

## Covariates

Information on the highest educational level, yearly family income level, and marital status was obtained from the Longitudinal Integration Database for Health Insurance and Labour Market study (LISA) database [32]. As LISA only provides information on individuals aged 16 or above, we used the parental socioeconomic status (SES) (i.e., highest educational level of the parents and yearly family income level) as a proxy for SES of individuals aged under 16. We defined family history of cancer as any cancer diagnosed before the index date, identified in the Cancer Register, among biological parents and full siblings. The burden of multiple somatic comorbidities was measured by the Charlson Comorbidity Index [33] (CCI), using diagnoses (excluding malignancies and metastatic solid tumors) in the National Patient Register. Substance use disorders were considered as an important covariate [18, 19] and commonly co-occur with or follow stress-related disorders [34, 35]. Therefore, we defined those who received a diagnosis of substance use disorders more than 3 months before the index date as having a history of substance use disorders, and those who received a diagnosis within 3 months before and 1 year after the diagnosis of stress-related disorders as having a comorbid substance use disorder. The most updated information before the index date for each covariate (except for history of substance use disorders) was used in the analysis. To detect the potential mediation role of behavior-related lifestyle factors, we identified smoking or alcohol-related morbidities as a group of diseases related to tobacco use or alcohol consumption that occurred during follow-up. ICD codes for all covariates are presented in Supplementary Table 1.

### Statistical analysis

We estimated the associations between stress-related disorders and risk of cancer incidence and mortality by hazard ratios (HRs) with 95% confidence intervals (CIs) derived from Cox regression models. Time since 1 year after the index date was used as the underlying time scale. The proportional hazard assumptions for Cox models were tested using Schoenfeld residuals and no violation was found.

In the population-matched cohort, all analyses were stratified by the matching identifiers (birth year and sex) and partially or fully adjusted for multiple potential confounders, including the highest educational level (< 9 years, 9–12 years, > 12 years, or unknown), family income level (top 20% [> 80% quantile], middle [between 20% and 80% quantiles], lowest 20% [< 20% quantile], or unknown), marital status (single [unmarried], married/cohabiting [married or registered partner], divorced/widowed [divorced/separated, widowed, divorced partner, or surviving partner], or unknown), family history of cancer (yes or no), CCI score, and history of substance use disorders (yes or no). In addition to the analysis for any stress-related disorder, we did separate analyses for PTSD, acute stress reaction, and adjustment disorder and other stress reactions. Also, we separately estimated the HR of cancer incidence and mortality for 13 cancer subtypes (according to the sites of origin) and 4 categories of cancer (according to shared etiologies). To account for multiple estimation, semi-Bayes shrinkage was used when analyzing the HRs for 13 site-specific cancers [36], which improves the plausibility and stability of estimates through attenuating individual associations toward the overall mean in proportion to their variance. Moreover, to further consider the potential confounding from familial factors, we repeated the main analyses in the sibling cohort using Cox regression models stratified by family identifiers and adjusted for birth year, sex, and all aforementioned covariates.

In stratification analyses, we calculated HRs by sex, age at index date (by tertile distribution: ≤28 years, 29–42 years, or ≥ 43 years), attained age (< 50 or ≥ 50 years), educational level (< 9 years, 9–12 years, or > 12 years), yearly family income level (lowest 20%, middle, or top 20%), time of follow-up (1–5 years, 6–10 years, or > 10 years), family history of cancer (yes or no), and history of substance use disorders (yes or no). We tested the differences of HRs by including an interaction term in the Cox regression models.

Following the analysis of cancer mortality, we further estimated the associations between stress-related disorders and cancer-related death after cancer diagnosis. We restricted the analysis to individuals (both patients with stress-related disorders and matched unexposed individuals or unaffected full siblings) who developed a cancer during the study and started the follow-up of these individuals from date of cancer diagnosis.

Given that psychological stress may induce subsequent unfavorable behaviors (e.g., smoking and alcohol abuse) which can further play a role on cancer development, in particular for smoking and alcohol-related cancers [7, 11], we conducted two additional analyses to examine the potential impact from behavior-related factors. First, we performed subgroup analyses by the presence of comorbid substance use disorders. The Wald test was used to examine the difference of HRs between subgroups. Second, we estimated the possible mediation effect of smoking or alcohol-related morbidities occurred after stress-related disorders using two models: (1) a multivariate logistic regression model for smoking or alcohol-related morbidities (mediator) conditioned on stress-related disorders (exposure) and all aforementioned covariates, and (2) a Cox regression model for smoking or alcohol-related cancer incidence or mortality (outcome) conditioned on stress-related disorders, smoking or alcohol-related morbidities, and all aforementioned covariates.

To address the concern about surveillance bias (i.e., patients with stress-related disorders tended to have closer contact with healthcare system than general population, leading to a higher likelihood of receiving a diagnosis of cancer as well as the possibility of death due to cancer), we additionally adjusted the frequency of healthcare visits for all individuals during the first year of follow-up in the Cox models. Also, in addition to the application of 1-year lag time in the main analyses, we repeated the analyses by excluding the first 2, 5, and 10 years of follow-up after cohort entry. To examine the impact of competing risk (i.e., deaths from other causes removed individuals from being at risk of death due to cancer), we further estimated the relative risk of cancer mortality by subhazard ratios (SHRs) based on the modified Cox model developed by Fine and Gray [37]. All analyses were conducted in SAS, version 9.4 (SAS Institute Inc). A 2-sided *P* < 0.05 was considered statistically significant. This cohort study was approved by the Regional Ethical Review Board in Stockholm, Sweden. The requirement for informed consent was waived for the register-based studies in Sweden.

## Results

In total, we included 167,836 exposed individuals, 1,631,801 matched unexposed individuals and 179,209 unaffected full siblings (Fig. 1). The median follow-up time was 7.13 (interquartile range [IQR] 3.09–11.92) and 7.27 years (IQR 3.16–12.21) for exposed and unexposed individuals, accumulating 16,386,919 person-years at risk in the population-matched cohort. With a median follow-up of 7.34 (IQR 3.21–12.12) and 7.82 years (IQR 3.49–13.10) for exposed individuals and their unaffected full siblings, 2,666,050 person-years were accumulated in the sibling cohort (Table 1). In the population-matched cohort, the mean age at stress-related disorder diagnosis was 36.57 years (standard deviation [SD] 14.16) and 38.08% of the exposed individuals were male. While there was little difference in family history of cancer, history of substance use disorders was more prevalent among exposed individuals than among unexposed individuals or their unaffected full siblings. Also, exposed individuals were more likely to be divorced or widowed, have lower family income level but higher burden of baseline somatic comorbidities, and have smoking or alcohol-related morbidities diagnosed during follow-up, both in the population-matched cohort and sibling cohort (Table 1).

**Table 1:** Baseline characteristics and follow-up data of study participants

Characteristics	Population-matched cohort		Sibling cohort	
	**Exposed individuals (N = 167,836)**	**Matched unaffected individuals (N = 1,631,801)**	**Exposed individuals (N = 101,493)**	**Unaffected full siblings (N = 179,209)**
**Age at index date, mean ± SD, years**	36.57 ± 14.16	36.21 ± 13.96	36.68 ± 13.67	37.62 ± 14.80
**Follow-up time (for cancer incidence), median (IQR), years**	7.13 (3.09–11.92)	7.27 (3.16–12.21)	7.34 (3.21–12.12)	7.82 (3.49–13.10)
**Follow-up time (for cancer mortality), median (IQR), years**	7.28 (3.17–12.11)	7.45 (3.24–12.44)	7.51 (3.30-12.31)	7.97 (3.61–13.39)
**Sex, N (%)**				
Male	63,920 (38.08)	624,287 (38.26)	38,735 (38.17)	92,107 (51.40)
Female	103,916 (61.92)	1,007,514 (61.74)	62,758 (61.83)	87,102 (48.60)
**Educational level, years, N (%)**				
< 9	6133 (3.65)	51,173 (3.14)	3517 (3.47)	10,083 (5.63)
9–12	111,806 (66.62)	971,908 (59.56)	68,945 (67.93)	116,585 (65.06)
> 12	39,148 (23.33)	505,262 (30.96)	25,865 (25.48)	45,756 (25.53)
Unknown	10,749 (6.40)	103,458 (6.34)	3166 (3.12)	6785 (3.78)
**Marital status, N (%)**				
Single	91,174 (54.32)	883,724 (54.16)	54,999 (54.19)	87,762 (48.97)
Married or cohabiting	40,538 (24.15)	559,519 (34.29)	25,496 (25.12)	63,594 (35.49)
Divorced or widowed	30,957 (18.44)	137,084 (8.40)	18,433 (18.16)	19,167 (10.70)
Unknown	5167 (3.09)	51,474 (3.15)	2565 (2.53)	8686 (4.84)
**Yearly family income level, N (%)**				
Lowest 20%	55,764 (33.23)	297,714 (18.24)	26,750 (26.36)	29,668 (16.55)
Middle	85,393 (50.88)	949,489 (58.19)	56,504 (55.67)	109,195 (60.93)
Top 20%	18,174 (10.83)	297,883 (18.25)	15,804 (15.57)	34,928 (19.49)
Unknown	8505 (5.06)	86,715 (5.32)	2435 (2.40)	5418 (3.03)
**Charlson Comorbidity Index, N (%)**				
0	133,457 (79.52)	1,417,180 (86.85)	81,682 (80.48)	150,913 (84.21)
1	26,462 (15.77)	174,958 (10.72)	15,235 (15.01)	22,533 (12.57)
≥ 2	7917 (4.72)	39,663 (2.43)	4576 (4.51)	5763 (3.22)
**Family history of cancer, N (%)**				
Yes	42,291 (25.20)	430,549 (26.38)	27,798 (27.39)	52,258 (29.16)
No	125,545 (74.80)	1,201,252 (73.62)	73,695 (72.61)	126,951 (70.84)
**History of substance use disorders**^**a**^, **N (%)**				
Yes	19,653 (11.71)	40,929 (2.51)	10,923 (10.76)	7701 (4.30)
No	148,183 (88.29)	1,590,872 (97.49)	90,570 (89.24)	171,508 (95.70)
**Comorbid substance use disorders**^**b**^, **N (%)**				
Yes	10,758 (6.41)	-	6289 (6.20)	-
No	157,078 (93.59)	-	95,204 (93.80)	-
**Type of stress**-**related disorders, N (%)**				
PTSD	12,613 (7.52)	-	7195 (7.09)	-
Acute stress reaction	72,970 (43.48)	-	44,044 (43.40)	-
Adjustment disorder and other stress reactions	76,738 (45.72)	-	46,956 (46.27)	-
**New onset morbidity during follow-up (for cancer incidence)**				
**Smoking-related morbidity**^**c**^, **N (%)**				
Yes	9328 (5.56)	46,079 (2.82)	5583 (5.50)	8012 (4.47)
No	158,508 (94.44)	1,585,722 (97.18)	95,910 (94.50)	171,197 (95.53)
**Alcohol-related morbidity**^**d**^, **N (%)**				
Yes	16,166 (9.63)	26,053 (1.60)	9598 (9.46)	4984 (2.78)
No	151,670 (90.37)	1,605,748 (98.40)	91,895 (90.54)	174,225 (97.22)
**New onset morbidity during follow-up (for cancer mortality)**				
**Smoking-related morbidity**^**c**^, **N (%)**				
Yes	9898 (5.90)	49,747 (3.05)	5907 (5.82)	8629 (4.82)
No	157,938 (94.10)	1,582,054 (96.95)	95,586 (94.18)	170,580 (95.18)
**Alcohol-related morbidity**^**d**^, **N (%)**				
Yes	16,284 (9.70)	26,544 (1.63)	9666 (9.52)	5075 (2.83)
No	151,552 (90.30)	1,605,257 (98.37)	91,827 (90.48)	174,134 (97.17)

Abbreviations: N, number; SD, standard deviation; IQR, interquartile range

^a^ First diagnosis of a substance use disorder, occurred > 3 months before index date

^b^ New onset substance use disorders that was diagnosed between 3 months before and 1 year after index date

^c^ Including COPD and coronary heart diseases

^d^ Including alcohol abuse and alcoholic liver cirrhosis

During up to 35 years of follow-up, we observed 6,918 and 65,742 incident cancer cases (crude incidence rate: 4.64 and 4.41 per 1000 person-years) as well as 2,006 and 15,756 cancer deaths (crude mortality rate: 1.32 and 1.04 per 1000 person-years) among the exposed and unexposed individuals in the population-matched cohort. In the sibling cohort, 4,073 and 8,598 incident cancer cases (crude incidence rate: 4.43 and 4.92 per 1000 person-years) as well as 1,136 and 2,377 cancer deaths (crude mortality rate: 1.21 and 1.33 per 1000 person-years) were observed in exposed individuals and their unaffected full siblings (Table 2). The proportion of advanced stage cancers was found to be higher in exposed individuals than unexposed individuals (Supplementary Table 2). In the fully adjusted Cox model, we found an increased risk of cancer incidence among exposed individuals, compared with unexposed individuals (HR = 1.03, 95% CI 1.01–1.06; Table 2). Among different stress-related disorders, the association was merely observed for adjustment disorder and other stress reactions (HR = 1.04, 95% CI 1.01–1.09). We found a more pronounced association between stress-related disorders and cancer mortality (HR = 1.13, 95% CI 1.07–1.18), especially for PTSD (HR = 1.35, 95% CI 1.10–1.66). However, when comparing exposed individuals with their unaffected full siblings, we observed null associations for cancer incidence and mortality (incidence: HR = 1.03, 95% CI 0.99–1.08; mortality: HR = 1.09, 95% CI 1.00-1.19), except for an increased risk of cancer mortality for acute stress reaction (HR = 1.16, 95% CI 1.02–1.33).

**Table 2:** Hazard ratios (HRs) with 95% confidence intervals (CIs) for cancer incidence and mortality among patients with stress-related disorders, compared with their matched unexposed individuals or unaffected full siblings

	Model information	Cancer incidence		Cancer mortality	
		**No of cases (incidence** ^**a**^ **) in exposed/unexposed individuals**	**HR (95% CI)**	**No of cases (mortality** ^**a**^ **) in exposed/unexposed individuals**	**HR (95% CI)**
**Population-matched cohort**	Sex, birth year	6918 (4.64)/65,742 (4.41)	1.07 (1.05–1.10)	2006 (1.32)/15,756 (1.04)	1.34 (1.28–1.41)
	As above + educational level, family income level, marital status		1.06 (1.03–1.09)		1.23 (1.17–1.29)
	As above + family history of cancer		1.06 (1.03–1.09)		1.23 (1.17–1.29)
	As above + Charlson comorbidity index		1.05 (1.02–1.08)		1.20 (1.14–1.26)
	As above + history of substance use disorders		1.03 (1.01–1.06)		1.13 (1.07–1.18)
	Full adjusted HRs for subtypes of stress-related disorders				
	PTSD	353 (3.89)/3607 (4.02)	0.95 (0.85–1.07)	117 (1.27)/817 (0.89)	1.35 (1.10–1.66)
	Acute stress reactions	2836 (4.53)/26,869 (4.30)	1.02 (0.98–1.07)	829 (1.30)/6284 (0.99)	1.13 (1.05–1.23)
	Adjustment disorder and other stress reactions	2989 (4.79)/27,667 (4.47)	1.04 (1.01–1.09)	760 (1.19)/6223 (0.99)	1.06 (0.98–1.15)
**Sibling cohort**	Sex, birth year	4073 (4.43)/8598 (4.92)	1.04 (1.00-1.09)	1136 (1.21)/2377 (1.33)	1.21 (1.12–1.31)
	As above + educational level, family income level, marital status		1.04 (1.00-1.09)		1.15 (1.06–1.25)
	As above + Charlson comorbidity index		1.04 (1.00-1.09)		1.14 (1.05–1.24)
	As above + history of substance use disorders		1.03 (0.99–1.08)		1.09 (1.00-1.19)
	Full adjusted HRs for subtypes of stress-related disorders				
	PTSD	206 (3.76)/448 (4.42)	1.01 (0.83–1.23)	63 (1.13)/138 (1.34)	1.00 (0.69–1.46)
	Acute stress reactions	1699 (4.40)/3599 (4.91)	1.04 (0.97–1.12)	489 (1.25)/993 (1.33)	1.16 (1.02–1.33)
	Adjustment disorder and other stress reactions	1750 (4.51)/3558 (4.99)	1.03 (0.96–1.10)	435 (1.10)/901 (1.24)	1.08 (0.95–1.24)

^a^ Per 1,000 person years

The stratification analyses in the population-based comparison showed that the associations between stress-related disorders and cancer incidence and mortality did not differ by sex, attained age, yearly family income level, time of follow-up, or family history of cancer (Supplementary Tables 3 and 4). However, the magnitude of associations was greater among individuals without a history of substance use disorders, compared with those with such a history (incidence: HR = 1.05, 95% CI 1.02–1.08 vs. 1.03, 95% CI 0.82–1.28, *P*_interaction_<0.001; mortality: HR = 1.16, 95% CI 1.10–1.23 vs. 0.89, 95% CI 0.62–1.28, *P*_interaction_<0.001). We also observed a stronger association between stress-related disorders and cancer mortality among younger individuals (≤ 28 years: HR = 1.25, 95% CI 0.98–1.60 vs. ≥ 43 years: 1.08, 95% CI 1.02–1.14, *P*_interaction_=0.02), and individuals with higher educational level (> 12 years: HR = 1.36, 95% CI 1.17–1.58 vs. < 9 years: 1.03, 95% CI 0.90–1.19, *P*_interaction_=0.03).

**Fig. 2:**
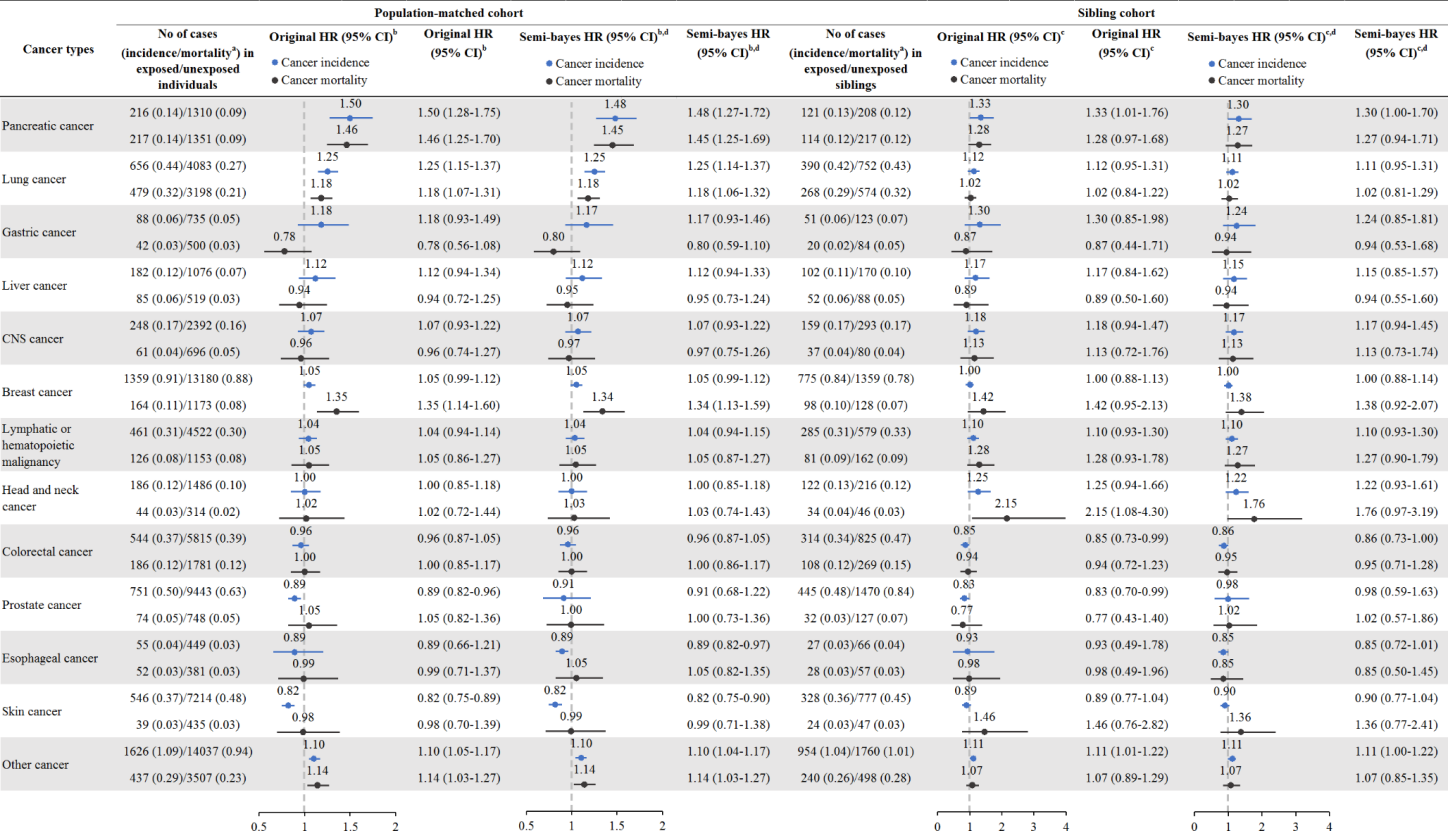
**Hazard ratios (HR) and 95% confidence intervals (CIs) for cancer incidence and mortality among patients with stress-related disorders compared with matched unexposed individuals or unaffected full siblings, before and after semi-Bayes shrinkage, by different sites of cancer** ^a^ Per 1000 person years ^b^ Cox regression models were stratified by matching identifiers (birth year and sex) and adjusted for educational level, family income level, marital status, family history of specific cancer, Charlson comorbidity index, and history of substance use disorders ^c^ Cox regression models were stratified by family identifiers and adjusted for birth year, sex, educational level, family income level, marital status, Charlson comorbidity index, and history of substance use disorders ^d^ For semi-Bayes shrinkage, we specified true population variance of 0.17 and 0.28 for cancer incidence and mortality, implying a prior expectation that 95% of the HRs would fall within a 5-fold range and 8-fold range, respectively

Figure 2 shows the HRs (before and after semi-Bayes shrinkage) for 13 subtypes of cancer in the population-matched cohort and sibling cohort. For cancer incidence, the strongest estimates were observed for pancreatic cancer (original HR = 1.50, 95% CI 1.28–1.75; semi-Bayes HR = 1.48, 95% CI 1.27–1.72) and lung cancer (original HR = 1.25, 95% CI 1.15–1.37; semi-Bayes HR = 1.25, 95% CI 1.14–1.37). We also observed an elevated risk of cancer mortality for pancreatic cancer (original HR = 1.46, 95% CI 1.25–1.70; semi-Bayes HR = 1.45, 95% CI 1.25–1.69), lung cancer (original HR = 1.18, 95% CI 1.07–1.31; semi-Bayes HR = 1.18, 95% CI 1.06–1.32), and breast cancer (original HR = 1.35, 95% CI 1.14–1.60; semi-Bayes HR = 1.34, 95% CI 1.13–1.59). Analysis for 4 categories of cancer with shared etiologies corroborated the results for site-specific cancer (Table 3), where we found higher risk of smoking or alcohol-related cancers (incidence: HR = 1.18, 95% CI 1.11–1.24; mortality: HR = 1.20, 95% CI 1.12–1.29), as well as an increased risk of hormone-related cancer mortality (HR = 1.20, 95% CI 1.06–1.35). When comparing with unaffected full siblings, we observed generally null results for either the site-specific cancer or categories of cancer (Fig. 2; Table 3).

**Table 3:** Hazard ratios (HRs) and 95% confidence intervals (CIs) for cancer incidence and mortality among patients with stress-related disorders, compared with their matched unexposed individuals or unaffected full siblings, by different types of cancer

	Cancer types	Cancer incidence		Cancer mortality	
		**No of cases (incidence** ^**a**^ **) in exposed/unexposed individuals**	**HR (95% CI)**	**No of cases (mortality** ^**a**^ **) in exposed/unexposed individuals**	**HR (95% CI)**
**Population-matched cohort** ^**b**^	Hematological malignancy	461 (0.31)/4522 (0.30)	1.04 (0.94–1.14)	126 (0.08)/1153 (0.08)	1.05 (0.86–1.27)
	Hormone-related cancer	2418 (1.62)/25,722 (1.73)	1.00 (0.95–1.04)	316 (0.21)/2625 (0.17)	1.20 (1.06–1.35)
	Immune-related cancer	1163 (0.78)/12,239 (0.82)	0.94 (0.88-1.00)	253 (0.17)/2055 (0.14)	1.01 (0.88–1.17)
	Smoking or alcohol-related cancer	1658 (1.11)/11,506 (0.77)	1.18 (1.11–1.24)	964 (0.63)/6422 (0.42)	1.20 (1.12–1.29)
**Sibling cohort** ^**c**^	Hematological malignancy	285 (0.31)/579 (0.33)	1.10 (0.93–1.30)	81 (0.09)/162 (0.09)	1.28 (0.93–1.78)
	Hormone-related cancer	1396 (1.52)/3144 (1.80)	0.98 (0.91–1.06)	174 (0.19)/343 (0.19)	1.04 (0.82–1.31)
	Immune-related cancer	691 (0.75)/1437 (0.82)	0.98 (0.88–1.08)	160 (0.17)/291 (0.16)	1.18 (0.92–1.50)
	Smoking or alcohol-related cancer	967 (1.05)/1886 (1.08)	1.12 (1.00-1.23)	541 (0.58)/1097 (0.61)	1.10 (0.96–1.25)

^a^ Per 1,000 person years

^b^ Cox regression models were stratified by matching identifiers (birth year and sex) and adjusted for educational level, family income level, marital status, family history of specific cancer type, Charlson comorbidity index, and history of substance use disorders

^c^ Cox regression models were stratified by family identifiers and adjusted for birth year, sex, educational level, family income level, marital status, Charlson comorbidity index, and history of substance use disorders

An overall association of stress-related disorders with risk of cancer-related death after cancer diagnosis was observed in the population-based comparison (HR = 1.19, 95% CI 1.10–1.28) as well as in the between-sibling comparison (HR = 1.24, 95% CI 1.02–1.51; Supplementary Table 5).

In the population-matched cohort, the presence of comorbid substance use disorders was associated with further elevated risk of smoking or alcohol-related cancers (incidence: *P*_difference_<0.001; mortality: *P*_difference_<0.001; Supplementary Table 6). However, this pattern was not observed in the between-sibling comparison (incidence: *P*_difference_=0.26; mortality: *P*_difference_=0.15). In addition, the mediation analysis in the population-matched cohort revealed that alcohol-related morbidities occurred during follow-up mediated 23.80% and 41.78% of the observed associations between stress-related disorders and smoking or alcohol-related cancer incidence and mortality, respectively (Table 4). Smoking-related morbidities mediated the associations to a smaller extent (mediated 0.06% and 4.63% of the observed associations). The mediation analysis was not performed in the sibling cohort due to the null association between stress-related disorders and smoking or alcohol-related cancers.

**Table 4:** Adjusted direct and indirect associations of stress-related disorders on smoking or alcohol-related cancer incidence and mortality mediated via new onset of smoking or alcohol-related morbidities during follow-up, in the population-matched cohort^a^

	Smoking or alcohol-related cancer incidence	Smoking or alcohol-related cancer mortality
	**HR (95% CI)**	**HR (95% CI)**
**Smoking-related morbidities**		
Total effect	1.173 (1.112,1.239)	1.150 (1.071,1.235)
Direct effect	1.173 (1.112,1.238)	1.143 (1.064,1.228)
Indirect effect	1.000 (1.000,1.001)	1.006 (1.005,1.007)
Proportion mediated, %	0.06	4.63
**Alcohol-related morbidities**		
Total effect	1.154 (1.094,1.218)	1.152 (1.073,1.237)
Direct effect	1.118 (1.058,1.181)	1.089 (1.013,1.170)
Indirect effect	1.033 (1.026,1.040)	1.058 (1.048,1.070)
Proportion mediated, %	23.80	41.78

^a^ Adjusted for matching variables (birth year and sex), as well as educational level, family income level, marital status, family history of cancer, Charlson comorbidity index, and history of substance use disorders

The sensitivity analyses in the population-matched cohort showed that additionally adjusting for frequency of healthcare visits during the first year of follow-up did not change the results (Supplementary Table 7). The association between stress-related disorders and cancer incidence diminished when extending the lag time to 2, 5, or 10 years, whereas the association between stress-related disorders and cancer mortality remained (Supplementary Table 8). The observed association between acute stress reaction and cancer mortality in the population-matched cohort showed generally unchanged results after adjusting for frequency of healthcare visits or extending the lag period, while then attenuated toward null in the sibling cohort (Supplementary Table 9). The observed associations for cancer mortality after accounting for competing risk was similar, although slightly weaker, compared with the HRs of the main analyses (Supplementary Table 10).

## Discussion

In this nationwide population-based and sibling-controlled cohort study, we observed a weak association between stress-related disorders and cancer risk and mortality, which was mainly explained by familial confounding. We also found that the excess risks in the incidence and mortality of smoking or alcohol-related cancers (e.g., pancreatic and lung cancers) among individuals with stress-related disorders were partially mediated by the presence of substance abuse, primarily alcohol-related morbidities during follow-up.

Considering the small effect size noted in the population-matched cohort, which attenuated to null in the analyses of between-sibling comparison and prolonged lag periods, the link between stress-related disorders and overall cancer incidence is weak, and explained by familial factors. In line with our findings, two register-based Danish cohort studies reported null associations of PTSD or adjustment disorder with overall cancer incidence [18, 19]. Another large-scale study, using a sample of 490,106 individuals in the UK, also indicated no associations between PTSD and the risk of prostate, colorectal and breast cancers [20]. In contrast, we found a moderate association between stress-related disorders and cancer mortality in the population-matched cohort. This association was however significantly attenuated in the between-sibling comparison. Most existing evidence on the role of stress-related disorders on cancer mortality suggested inconsistent findings, reporting both positive and null associations [17, 38, 39]. These prior studies have a specific focus on PTSD [17, 38, 39] and lung cancer [39]. In addition, this association may be explained by residual confounding (e.g., factors shared within families), while none of the existing studies had stringently controlled for familial factors [17, 38, 39]. To the best of our knowledge, the present study is the first to provide a comprehensive assessment on the associations between all stress-related disorders and multiple types of cancer, in terms of both cancer incidence and mortality. Our finding of a positive association between stress-related disorders and cancer risk and mortality in the population-based comparison, which then attenuated towards null in the between-sibling comparison, indicates that such association is largely attributed to familial confounding. Yet, our finding of an increased risk of cancer-related death after the cancer diagnosis among individuals with stress-related disorders, both in the population- and between-sibling comparisons, is in line with earlier research indicating a role of psychiatric disorders in cancer-related progression [40, 41].

The finding of an elevated risk of smoking or alcohol-related cancer incidence and mortality in the population-matched cohort, especially pancreatic and lung cancers, corroborated with a recent Danish cohort study reporting a positive association between adjustment disorder and smoking or alcohol-related cancers [19]. Based on the subgroup and mediation analysis, we found that the risk elevation in incidence and mortality of smoking or alcohol-related cancers was mainly attributable to the presence of comorbid substance abuse and partially mediated by the onset of alcohol-related morbidities after stress-related disorders, supporting the notion that the stress-related unfavorable behaviors might mediate the increased risk of cancer development and death following stress exposure [1, 7]. By contrast, in the between-sibling comparison, null association for smoking or alcohol-related cancers was observed and the presence of comorbid substance use disorders did not significantly modify the risk of smoking or alcohol-related cancers, suggesting that siblings might share these behavioral risk factors.

The major strength of our study is the population-based cohort design with a complete follow-up of up to 35 years, as well as the between-sibling comparison to additionally address the concern about familial confounding. The registration and diagnosis of stress-related disorders and cancer incidence and death were collected prospectively and independently, which minimized the risk of information bias. The large sample size also allowed us to perform detailed subgroup analyses, including exploring risks of specific cancer sites and cancer groups. In addition, we were able to consider for a wide range of confounding and mediating factors, including sociodemographic factors and somatic and psychiatric comorbidities.

This study also has some limitations. One concern was reverse causality, i.e., PTSD and other stress-related disorders might be subsequent to pre-clinical cancer symptoms [27]. In our analysis, we applied multiple lag periods to ensure the temporal order of stress-related disorders and cancer diagnosis. The results diminished in these analyses, especially for cancer incidence. Second, although the PTSD diagnosis in the National Patient Register was validated to a high degree [42], the validity of other stress-related disorders was not documented and might have introduced bias in the analysis. However, such misclassification, if any, should most likely have been non-differential and led to an attenuated magnitude of the studied association. Third, there was a potential surveillance bias in the study (i.e., patients with stress-related disorders may have a higher likelihood of receiving a diagnosis of cancer due to the frequent healthcare visits). However, we obtained generally similar results in the sensitivity analyses where we further adjusted for healthcare visits during the first year after cohort entry. Fourth, although small, a proportion of the sibling controls received a diagnosis of stress-related disorders during follow-up. Although we included them to the exposed group after the diagnosis, they might have demonstrated symptoms for stress-related disorders already before the diagnosis. Similarly, although vast majority of the sibling controls did not formally receive a diagnosis of stress-related disorders, they might have suffered from symptoms related to stress-related disorders as traumatic events of one person likely affect the whole family. Both of these might have led to conservative estimates in the sibling analysis. Last, we had no direct measurement of stress-related behaviors other than comorbid substance abuse and smoking- and alcohol-related morbidities subsequent to stress-related disorders. Future research with detailed data on lifestyle, ideally with longitudinal measurements after the diagnosis of stress-related disorders, is warranted.

In conclusion, our findings suggest that the modestly elevated risks of cancer incidence and mortality among individuals with stress-related disorders are mainly driven by familial factors. The contributing role of substance abuse, primarily alcohol-related morbidities, on the associations of stress-related disorders with smoking or alcohol-related cancer incidence and mortality also indicated the role of behavioral risk factors on the cancer development.

## Electronic supplementary material

Below is the link to the electronic supplementary material.

Supplementary Material 1

Supplementary Material 2

## Data Availability

The data are not publicly available due to Swedish laws. More information regarding the data access to SIMSAM can be found at: https://simsam.nu.
