# Supplementary material for: Stress-related disorders and subsequent cancer risk and mortality: a population-based and sibling-controlled cohort study in Sweden
Source: Eur J Epidemiol. 2022 Aug 13;37(9):947–58. doi: 10.1007/s10654-022-00898-x (PMC9529708; doi:10.1007/s10654-022-00898-x)
Supplement: Supplementary file 2 — Supplementary Material 2 [file 10654_2022_898_MOESM2_ESM.docx]

**Supplementary Table 1. International classification of diseases (ICD) codes for exposure, outcome and covariates**

|  | **ICD10** | **ICD9** | **ICD8** | **ICD7** |
| --- | --- | --- | --- | --- |
| **All stress-related disorder** | F43 | 308, 309 | 307 |  |
| PTSD | F43.1 | 309B |  |  |
| Acute stress reaction | F43.0 | 308, 309A |  |  |
| Adjustment disorder | F43.2 | 309X |  |  |
| Other stress reactions | F43.8 | 309X |  |  |
|  | F43.9 | 309X |  |  |
| **Alcohol-related morbidity** | | | | |
| Alcohol abuse | F10, G31.2, G62.1, G72.1, I42.6, K29.2, K70, K86.0, Q35.4; R78.9, T51.0, T51.8, T51.9, X65, Y15, Y57.3, Y90, Y91, Z50.2, Z71.4, Z71.2 | 291, 303, 305A, 357F, 425F, 535D,571A–D, 760W, 790D, 977D, 980A, 980X, V97B | 261.00, 262.00, 291, 303, 571.00, 571.01, 979, 980.00, 980.01, 980.98, 980.99 |  |
| Alcoholic liver cirrhosis | K70.3, K74.6 | 571C, 571D | 571.00 |  |
| **Smoking-related morbidity** | | | | |
| Nicotine dependence | F17 |  |  |  |
| COPD | J41-J44 | 491, 492, 496 | 491, 492 |  |
| Coronary heart diseases | I20-I25 | 410-414 | 410-414 |  |
| **Substance use disorder** | F10-F19 | 291, 303, 304, 305A, 305X | 291, 303, 304 |  |
| **Cancer types according to site** | | | | |
| All cancers |  |  |  | 140-209 |
| Gastric cancer |  |  |  | 151 |
| Esophageal cancer |  |  |  | 150 |
| Colorectal cancer |  |  |  | 153, 154 |
| Skin cancer |  |  |  | 190, 191 |
| Liver cancer |  |  |  | 155, 156 |
| Pancreatic cancer |  |  |  | 157 |
| Lung cancer |  |  |  | 162, 163 |
| Breast cancer |  |  |  | 170 |
| Prostate cancer |  |  |  | 177 |
| CNS cancer |  |  |  | 193 |
| Head and neck cancer |  |  |  | 140-148, 160, 161 |
| Lymphatic or hematopoietic malignancies |  |  |  | 200-207 |
| Other cancers |  |  |  | 140-209, excluding above |
| **Cancer types according to etiology** | | | | |
| Hematological malignancies |  |  |  | 200-207 |
| Hormone-related cancers |  |  |  | 170, 172, 175, and 177 |
| Immune-related cancers |  |  |  | 155, 171, 190, 191, 200-202, 204 |
| Smoking or alcohol-related cancers |  |  |  | 140, 141, 143-150, 155, 157, 160-162, 180, 181 |
| **Cancer deaths according to site** | | | | |
| All cancer death | C00-C97 | 140-209 | 140-209 |  |
| Gastric cancer | C16 | 151 | 151 |  |
| Esophageal cancer | C15 | 150 | 150 |  |
| Colorectal cancer | C18-C21 | 153, 154 | 153, 154 |  |
| Skin cancer | C43-C44 | 172, 173 | 172, 173 |  |
| Liver cancer | C22 | 155, 156 | 155, 156 |  |
| Pancreatic cancer | C25 | 157 | 157 |  |
| Lung cancer | C33-C34 | 162 | 162 |  |
| Breast cancer | C50 | 174 | 174 |  |
| Prostate cancer | C61 | 185 | 185 |  |
| CNS cancer | C70-C72 | 191, 192 | 191, 192 |  |
| Head and neck cancer | C00-C14, C30, C32, C69 | 140-149, 160, 161, 190 | 140-149, 160, 161, 190 |  |
| Lymphatic or hematopoietic malignancies | C81-C85, C88, C90-C96 | 200-209 | 200-209 |  |
| Other cancers | C00-C97, excluding above | 140-209, excluding above | 140-209, excluding above |  |
| **Cancer deaths according to etiology** | | | | |
| Hematological malignancies | C81-C85, C88, C90-C96 | 200-209 | 200-209 |  |
| Hormone-related cancers | C50, C54, C56, C570-574, C61 | 174, 182, 183, 185 | 174, 182, 183, 185 |  |
| Immune-related cancers | C21-C22, C43-C44, C46, B210, C51, C53, C81-C85, C91-C95 | 155, 156, 172, 173, 180, 200-202, 204-208 | 155, 156, 172, 173, 180, 200-202, 204-208 |  |
| Smoking or alcohol-related cancers | C00-C06, C09-C15, C22, C25, C30-C34, C64, C67 | 140, 141, 143-150, 155, 157, 160-162, 188, 189 | 140, 141, 143-150, 155, 157, 160-162, 188, 189 |  |

**Supplementary Table 2. Cancer stage among exposed individuals and their matched unexposed individuals and unaffected full siblings**

| **Cancer stage^a^** | **Population-matched cohort** | | **Sibling cohort** | |
| --- | --- | --- | --- | --- |
|  | **Exposed individuals (N=6,918)** | **Unexposed individuals (N=65,742)** | **Exposed individuals (N=4,073)** | **Unaffected full siblings (N=8,598)** |
| Localized limited | 1204 (17.40) | 11677 (17.76) | 729 (17.90) | 1370 (15.93) |
| Localized advanced | 191 (2.76) | 1878 (2.86) | 121 (2.97) | 267 (3.11) |
| Regional spread | 555 (8.02) | 4791 (7.29) | 326 (8.00) | 672 (7.82) |
| Distant metastasis | 534 (7.72) | 4508 (6.86) | 296 (7.27) | 704 (8.19) |
| Unknown | 4026 (58.20) | 38785 (59.00) | 2352 (57.75) | 5060 (58.85) |
| Not applicable^b^ | 408 (5.90) | 4103 (6.24) | 249 (6.11) | 525 (6.11) |

Abbreviations: N*,* number.

^a^ Cancer stage was defined by European Network of Cancer Registries Condensed TNM Scheme: localized limited (T-localized/N0/M0), localized advanced (T-advanced/N0/M0), Regional spread (any T/N+/M0), distant metastasis (any T/any N/M+), or unknown stage. Since cancer stage has been collected since 2004, a large proportion of cancer were classified as unknown due to no records for the stage.

^b^ Hematological malignancies were not applicable to the staging system.

**Supplementary Table 3. Number of cases and crude incidence/mortality rates (per 1,000 person-years) for cancer among patients with stress-related disorders, compared with their matched unexposed individuals, by different characteristics**

| **Characteristics** | **Cancer incidence** | | | | **Cancer mortality** | | | |
| --- | --- | --- | --- | --- | --- | --- | --- | --- |
|  | **All** | **PTSD** | **Acute stress reaction** | **Adjustment disorder and other stress reactions** | **All** | **PTSD** | **Acute stress reaction** | **Adjustment disorder and other stress reactions** |
| **Sex** | | | | | | | | |
| Male | 2721 (4.72)/26822 (4.54) | 132 (4.85)/1250 (4.52) | 1213 (4.64)/11659 (4.37) | 1108 (4.84)/10813 (4.66) | 857 (1.46)/6849 (1.14) | 42 (1.51)/324 (1.15) | 382 (1.44)/2856 (1.05) | 319 (1.37)/2625 (1.11) |
| Female | 4197 (4.59)/38920 (4.33) | 221 (3.49)/2357 (3.79) | 1623 (4.44)/15210 (4.24) | 1881 (4.75)/16854 (4.36) | 1149 (1.23)/8907 (0.97) | 75 (1.17)/493 (0.78) | 447 (1.20)/3428 (0.94) | 441 (1.09)/3598 (0.91) |
| **Age at entry, years** | | | | | | | | |
| ≤28 | 542 (1.06)/5117 (1.01) | 27 (0.74)/287 (0.80) | 206 (0.98)/1872 (0.9) | 175 (0.90)/1715 (0.89) | 78 (0.15)/605 (0.12) | 2 (0.05)/19 (0.05) | 30 (0.14)/196 (0.09) | 20 (0.10)/162 (0.08) |
| 29-42 | 1866 (3.55)/17572 (3.30) | 95 (3.33)/924 (3.23) | 694 (3.11)/6632 (2.95) | 692 (3.22)/6173 (2.88) | 502 (0.94)/3527 (0.65) | 23 (0.79)/162 (0.56) | 159 (0.70)/1166 (0.51) | 168 (0.77)/1049 (0.48) |
| ≥43 | 4510 (9.95)/43053 (9.54) | 231 (9.01)/2396 (9.51) | 1936 (10.04)/18365 (9.55) | 2122 (9.87)/19779 (9.33) | 1426 (3.03)/11624 (2.47) | 92 (3.48)/636 (2.41) | 640 (3.20)/4922 (2.46) | 572 (2.56)/5012 (2.28) |
| **Attained age, years** | | | | | | | | |
| <50 | 1520 (0.69)/13883 (0.64) | 88 (0.42)/853 (0.42) | 611 (0.64)/5757 (0.61) | 672 (0.71)/5973 (0.64) | 225 (0.10)/1586 (0.07) | 8 (0.04)/77 (0.04) | 90 (0.09)/622 (0.07) | 94 (0.10)/627 (0.07) |
| ≥50 | 5248 (6.68)/48836 (6.52) | 256 (6.49)/2609 (6.94) | 2177 (6.88)/20053 (6.62) | 2249 (7.12)/20594 (6.85) | 1748 (2.14)/13613 (1.72) | 105 (2.57)/717 (1.80) | 725 (2.21)/5480 (1.72) | 657 (2.00)/5411 (1.71) |
| **Educational level, years** | | | | | | | | |
| <9 | 890 (11.83)/8510 (12.53) | 53 (10.29)/518 (12.31) | 423 (11.93)/3843 (12.48) | 382 (12.71)/3760 (13.14) | 363 (4.61)/3121 (4.35) | 30 (5.64)/201 (4.50) | 177 (4.76)/1407 (4.32) | 139 (4.41)/1352 (4.47) |
| 9-12 | 3916 (4.02)/34186 (3.95) | 215 (3.35)/2029 (3.67) | 1799 (3.95)/15359 (3.85) | 1777 (4.17)/15725 (4.08) | 1073 (1.08)/7902 (0.90) | 61 (0.94)/446 (0.79) | 507 (1.09)/3577 (0.88) | 461 (1.06)/3574 (0.91) |
| >12 | 1516 (5.46)/17056 (4.36) | 80 (5.13)/1031 (4.18) | 597 (5.10)/7529 (4.22) | 804 (5.70)/8030 (4.48) | 328 (1.15)/2792 (0.70) | 25 (1.57)/165 (0.65) | 140 (1.17)/1253 (0.69) | 153 (1.06)/1268 (0.69) |
| **Yearly family income level** | | | | | | | | |
| Lowest 20% | 2670 (4.52)/13277 (4.00) | 158 (3.97)/815 (4.02) | 1248 (4.44)/6079 (3.85) | 1150 (4.66)/5798 (4.16) | 913 (1.52)/4110 (1.22) | 53 (1.31)/239 (1.15) | 439 (1.53)/1895 (1.18) | 379 (1.51)/1787 (1.26) |
| Middle | 3316 (4.91)/41156 (4.75) | 180 (4.26)/2464 (4.53) | 1439 (4.77)/18419 (4.67) | 1620 (5.11)/18937 (4.83) | 812 (1.18)/9101 (1.03) | 61 (1.42)/534 (0.96) | 369 (1.20)/4101 (1.02) | 353 (1.09)/4082 (1.02) |
| Top 20% | 367 (4.72)/5471 (4.02) | 14 (3.28)/313 (2.99) | 147 (4.55)/2307 (3.90) | 205 (4.99)/2840 (4.30) | 50 (0.63)/659 (0.48) | 3 (0.69)/40 (0.38) | 21 (0.64)/276 (0.46) | 26 (0.62)/342 (0.51) |
| **Time of follow-up, years** | | | | | | | | |
| 0-5 | 2187 (2.67)/19672 (2.46) | 127 (2.05)/1151 (1.90) | 915 (2.57)/8447 (2.43) | 1108 (2.95)/9740 (2.67) | 391 (0.47)/2955 (0.37) | 25 (0.40)/138 (0.23) | 178 (0.50)/1299 (0.37) | 181 (0.48)/1442 (0.39) |
| 6-10 | 1739 (3.40)/15056 (3.13) | 75 (2.34)/810 (2.68) | 720 (3.27)/6335 (3.06) | 878 (3.76)/7433 (3.41) | 487 (0.93)/3566 (0.72) | 33 (1.01)/164 (0.53) | 215 (0.96)/1458 (0.68) | 217 (0.91)/1812 (0.80) |
| >10 | 2992 (7.18)/25832 (6.54) | 151 (7.38)/1379 (7.23) | 1201 (7.37)/9918 (6.50) | 1003 (7.28)/8494 (6.59) | 1128 (2.59)/7969 (1.89) | 59 (2.76)/439 (2.15) | 436 (2.57)/2965 (1.82) | 352 (2.51)/2543 (1.84) |
| **Family history of cancer** | | | | | | | | |
| Yes | 2468 (7.36)/24903 (6.59) | 125 (6.41)/1362 (6.41) | 1046 (7.27)/10446 (6.53) | 1141 (7.51)/11369 (6.86) | 696 (2.02)/5926 (1.52) | 44 (2.20)/322 (1.47) | 297 (2.01)/2471 (1.50) | 284 (1.82)/2603 (1.53) |
| No | 4450 (3.85)/40839 (3.67) | 228 (3.20)/2245 (3.27) | 1790 (3.71)/16423 (3.53) | 1848 (3.91)/16298 (3.60) | 1310 (1.12)/9830 (0.87) | 73 (1.01)/495 (0.71) | 532 (1.09)/3813 (0.81) | 476 (0.99)/3620 (0.79) |
| **History of substance use disorders^a^** | | | | | | | | |
| Yes | 823 (5.83)/1683 (6.66) | 42 (4.31)/78 (4.91) | 386 (5.62)/730 (6.38) | 309 (5.81)/772 (6.93) | 336 (2.34)/708 (2.75) | 15 (1.52)/31 (1.93) | 162 (2.32)/307 (2.64) | 111 (2.05)/320 (2.82) |
| No | 6095 (4.52)/64059 (4.37) | 311 (3.84)/3529 (4.00) | 2450 (4.39)/26139 (4.26) | 2680 (4.69)/26895 (4.43) | 1670 (1.21)/15048 (1.01) | 102 (1.24)/786 (0.87) | 667 (1.17)/5977 (0.96) | 649 (1.11)/5903 (0.95) |

^a^ First diagnosis of substance use disorders, occurred >3 months before index date.

**Supplementary Table 4. Hazard ratios (HRs) and 95% confidence intervals (CIs)^a^ for cancer incidence and mortality among patients with stress-related disorders, compared with their matched unexposed individuals, by different characteristics**

| **Characteristics** | **Cancer incidence** | | | | **Cancer mortality** | | | |
| --- | --- | --- | --- | --- | --- | --- | --- | --- |
|  | **All** | **PTSD** | **Acute stress reaction** | **Adjustment disorder and other stress reactions** | **All** | **PTSD** | **Acute stress reaction** | **Adjustment disorder and other stress reactions** |
| **Sex** | | | | | | | | |
| Male | 1.02 (0.98-1.06) | 1.06 (0.88-1.28) | 1.03 (0.97-1.10) | 1.01 (0.95-1.08) | 1.12 (1.03-1.20) | 1.26 (0.89-1.78) | 1.15 (1.03-1.29) | 1.06 (0.93-1.19) |
| Female | 1.04 (1.01-1.07) | 0.90 (0.78-1.04) | 1.02 (0.96-1.07) | 1.06 (1.01-1.12) | 1.14 (1.07-1.21) | 1.42 (1.10-1.84) | 1.12 (1.01-1.25) | 1.06 (0.95-1.17) |
| *P*_interaction_^b^ | 0.57 |  |  |  | 0.81 |  |  |  |
| **Age at entry, years** | | | | | | | | |
| ≤28 | 1.06 (0.97-1.16) | 0.90 (0.59-1.36) | 1.07 (0.93-1.25) | 1.04 (0.89-1.23) | 1.25 (0.98-1.60) | 0.87 (0.19-3.99) | 1.44 (0.97-2.14) | 1.15 (0.70-1.90) |
| 29-42 | 1.08 (1.02-1.13) | 1.06 (0.84-1.32) | 1.03 (0.95-1.12) | 1.12 (1.03-1.22) | 1.26 (1.13-1.39) | 1.41 (0.87-2.29) | 1.14 (0.95-1.36) | 1.39 (1.17-1.66) |
| ≥43 | 1.01 (0.98-1.04) | 0.92 (0.80-1.06) | 1.01 (0.96-1.06) | 1.03 (0.98-1.07) | 1.08 (1.02-1.14) | 1.35 (1.07-1.70) | 1.12 (1.03-1.22) | 0.98 (0.90-1.08) |
| *P*_interaction_^b^ | 0.19 |  |  |  | 0.02 |  |  |  |
| **Attained age, years** | | | | | | | | |
| <50 | 1.08 (1.02-1.14) | 1.04 (0.82-1.30) | 1.05 (0.96-1.15) | 1.10 (1.02-1.20) | 1.25 (1.07-1.44) | 0.93 (0.43-2.01) | 1.26 (1.00-1.59) | 1.34 (1.06-1.68) |
| ≥50 | 1.02 (0.99-1.05) | 0.92 (0.80-1.05) | 1.02 (0.98-1.07) | 1.03 (0.98-1.08) | 1.11 (1.05-1.17) | 1.35 (1.08-1.68) | 1.12 (1.03-1.22) | 1.03 (0.95-1.12) |
| *P*_interaction_^b^ | 0.15 |  |  |  | 0.06 |  |  |  |
| **Educational level, years** | | | | | | | | |
| <9 | 1.04 (0.95-1.14) | 1.10 (0.77-1.57) | 1.10 (0.97-1.26) | 0.99 (0.86-1.13) | 1.03 (0.90-1.19) | 2.01 (1.20-3.37) | 1.15 (0.94-1.41) | 0.83 (0.66-1.04) |
| 9-12 | 1.04 (1.00-1.08) | 0.94 (0.81-1.10) | 1.03 (0.97-1.09) | 1.05 (1.00-1.11) | 1.13 (1.05-1.21) | 1.16 (0.86-1.57) | 1.11 (1.00-1.23) | 1.13 (1.01-1.26) |
| >12 | 1.09 (1.02-1.16) | 1.02 (0.77-1.35) | 1.04 (0.94-1.16) | 1.11 (1.02-1.21) | 1.36 (1.17-1.58) | 2.12 (1.19-3.77) | 1.35 (1.07-1.71) | 1.25 (1.00-1.55) |
| *P*_interaction_^b^ | 0.68 |  |  |  | 0.03 |  |  |  |
| **Yearly family income level** | | | | | | | | |
| Lowest 20% | 0.98 (0.93-1.03) | 0.88 (0.72-1.09) | 1.01 (0.93-1.09) | 0.96 (0.88-1.04) | 1.03 (0.94-1.13) | 1.12 (0.78-1.60) | 1.08 (0.95-1.24) | 0.99 (0.86-1.13) |
| Middle | 1.06 (1.02-1.10) | 1.00 (0.85-1.17) | 1.05 (0.99-1.11) | 1.08 (1.02-1.14) | 1.19 (1.10-1.28) | 1.56 (1.16-2.12) | 1.23 (1.09-1.38) | 1.10 (0.98-1.23) |
| Top 20% | 1.09 (0.94-1.26) | 0.49 (0.20-1.18) | 1.27 (1.00-1.62) | 1.04 (0.86-1.26) | 1.23 (0.77-1.95) | NA | 1.19 (0.56-2.54) | 1.26 (0.69-2.31) |
| *P*_interaction_^b^ | 0.32 |  |  |  | 0.06 |  |  |  |
| **Time of follow-up, years** | | | | | | | | |
| 0-5 | 1.03 (0.98-1.07) | 1.08 (0.89-1.30) | 0.99 (0.93-1.07) | 1.05 (0.98-1.12) | 1.05 (0.94-1.18) | 1.51 (0.96-2.39) | 1.07 (0.91-1.27) | 1.01 (0.86-1.18) |
| 6-10 | 1.01 (0.96-1.06) | 0.82 (0.64-1.04) | 0.99 (0.91-1.07) | 1.02 (0.95-1.10) | 1.06 (0.96-1.17) | 1.73 (1.16-2.58) | 1.15 (0.99-1.34) | 0.92 (0.80-1.07) |
| >10 | 1.05 (1.01-1.10) | 0.92 (0.77-1.10) | 1.07 (1.01-1.15) | 1.06 (0.99-1.14) | 1.19 (1.12-1.28) | 1.17 (0.87-1.56) | 1.16 (1.04-1.30) | 1.20 (1.07-1.35) |
| *P*_interaction_^b^ | 0.07 |  |  |  | 0.08 |  |  |  |
| **Family history of cancer** | | | | | | | | |
| Yes | 1.02 (0.97-1.07) | 0.79 (0.64-0.98) | 1.04 (0.96-1.12) | 1.01 (0.94-1.08) | 1.08 (0.99-1.19) | 1.27 (0.86-1.87) | 1.10 (0.95-1.27) | 0.99 (0.86-1.14) |
| No | 1.03 (1.00-1.07) | 1.02 (0.88-1.18) | 1.01 (0.96-1.07) | 1.07 (1.01-1.13) | 1.14 (1.07-1.22) | 1.42 (1.07-1.86) | 1.14 (1.03-1.26) | 1.13 (1.01-1.25) |
| *P*_interaction_^b^ | 0.91 |  |  |  | 0.46 |  |  |  |
| **History of substance use disorders^c^** | | | | | | | | |
| Yes | 1.03 (0.82-1.28) | 3.29 (0.30-35.90) | 1.03 (0.75-1.41) | 0.98 (0.68-1.40) | 0.89 (0.62-1.28) | NA | 0.99 (0.60-1.66) | 0.58 (0.30-1.11) |
| No | 1.05 (1.02-1.08) | 0.95 (0.84-1.07) | 1.04 (1.00-1.09) | 1.06 (1.02-1.11) | 1.16 (1.10-1.23) | 1.37 (1.10-1.70) | 1.18 (1.08-1.28) | 1.10 (1.01-1.19) |
| *P*_interaction_^b^ | <0.001 |  |  |  | <0.001 |  |  |  |

^a^ Derived from Cox regression models, stratified by matching identifiers (birth year and sex) and adjusted for educational level, family income level, marital status, family history of cancer, Charlson comorbidity index, and history of substance use disorders.

^b^ The difference of HRs in subgroups was tested by including an interaction term in the Cox regression models.

^c^ First diagnosis of substance use disorders, occurred >3 months before index date.

NA indicates that the HR cannot be estimated due to few cases.

**Supplementary Table 5. Hazard ratios (HRs) and 95% confidence intervals (CIs) for risk of cancer-related death after cancer diagnosis among patients with stress-related disorders who developed cancer during the study, compared to their matched unexposed individuals and unaffected full siblings who also developed cancer during the study**

|  | **Cancer types** | **Cancer mortality** | |
| --- | --- | --- | --- |
|  |  | **No of cases (mortality^a^) in exposed/unexposed individuals** | **HR^b^ (95% CI)** |
| **Population-matched cohort** | Any cancer | 2006 (6.69)/15756 (4.98) | 1.19 (1.10-1.28) |
|  | Hematological malignancy | 126 (0.42)/1153 (0.36) | 1.52 (1.11-2.09) |
|  | Hormone-related cancer | 316 (1.05)/2625 (0.83) | 1.23 (0.98-1.53) |
|  | Immune-related cancer | 253 (0.84)/2055 (0.65) | 1.43 (1.12-1.82) |
|  | Smoking and alcohol-related cancer | 964 (3.21)/6422 (2.03) | 1.27 (1.13-1.43) |
| **Sibling cohort** | Any cancer | 1136 (6.46)/2377 (6.03) | 1.24 (1.02-1.51) |
|  | Hematological malignancy | 81 (0.46)/162 (0.41) | 1.24 (0.57-2.72) |
|  | Hormone-related cancer | 174 (0.99)/343 (0.87) | 1.53 (0.83-2.85) |
|  | Immune-related cancer | 160 (0.91)/291 (0.74) | 1.27 (0.68-2.35) |
|  | Smoking and alcohol-related cancer | 541 (3.07)/1097 (2.78) | 1.14 (0.87-1.51) |

^a^ Per 100 person years.

^b^ In the population-matched cohort, Cox regression models were stratified by matching identifiers (birth year and sex) and adjusted for educational level, family income level, marital status, family history of specific cancer type, Charlson comorbidity index, and history of substance use disorders. In the sibling cohort, Cox regression models were stratified by family identifiers and adjusted for birth year, sex, educational level, family income level, marital status, Charlson comorbidity index, and history of substance use disorders.

**Supplementary Table 6. Hazard ratios (HRs) and 95% confidence intervals (CIs) for cancer incidence and mortality among patients with stress-related disorders, compared with their matched unexposed individuals or unaffected full siblings, by the occurrence of comorbid substance use disorders**

|  | **Cancer types** | **Occurrence of comorbid substance use disorders** | **Cancer incidence** | | | **Cancer mortality** | | |
| --- | --- | --- | --- | --- | --- | --- | --- | --- |
|  |  |  | **No of cases (incidence^a^) in exposed/unexposed individuals** | **HR** | ***P*_difference_^b^** | **No of cases (mortality^a^) in exposed/unexposed individuals** | **HR** | ***P*_difference_^b^** |
| **Population-matched cohort^c^** | Any cancer | Yes | 498 (5.2)/4359 (4.35) | 1.27 (1.15-1.41) | <0.001 | 172 (1.76)/1094 (1.07) | 1.70 (1.43-2.02) | <0.001 |
|  |  | No | 6420 (4.61)/61383 (4.42) | 1.02 (0.99-1.04) |  | 1834 (1.29)/14662 (1.03) | 1.09 (1.04-1.15) |  |
|  | Hematological malignancy | Yes | 34 (0.35)/347 (0.35) | 1.02 (0.70-1.48) | 0.93 | 9 (0.09)/89 (0.09) | 1.07 (0.52-2.19) | 0.97 |
|  |  | No | 427 (0.31)/4175 (0.30) | 1.04 (0.94-1.15) |  | 117 (0.08)/1064 (0.07) | 1.05 (0.86-1.29) |  |
|  | Hormone-related cancer | Yes | 147 (1.53)/1683 (1.68) | 1.05 (0.88-1.26) | 0.53 | 22 (0.23)/179 (0.17) | 1.52 (0.94-2.45) | 0.32 |
|  |  | No | 2271 (1.63)/24039 (1.73) | 0.99 (0.95-1.04) |  | 294 (0.21)/2446 (0.17) | 1.18 (1.04-1.34) |  |
|  | Immune-related cancer | Yes | 82 (0.86)/796 (0.79) | 1.11 (0.87-1.41) | 0.15 | 20 (0.20)/166 (0.16) | 1.31 (0.80-2.16) | 0.30``` |
|  |  | No | 1081 (0.78)/11443 (0.82) | 0.92 (0.87-0.99) |  | 233 (0.16)/1889 (0.13) | 1.00 (0.86-1.16) |  |
|  | Smoking or alcohol-related cancer | Yes | 151 (1.58)/830 (0.83) | 1.81 (1.50-2.20) | <0.001 | 88 (0.90)/469 (0.46) | 1.93 (1.50-2.49) | <0.001 |
|  |  | No | 1507 (1.08)/10676 (0.77) | 1.13 (1.07-1.20) |  | 876 (0.62)/5953 (0.42) | 1.16 (1.07-1.25) |  |
| **Sibling cohort^d^** | Any cancer | Yes | 293 (5.09)/542 (4.66) | 1.27 (1.06-1.51) | 0.02 | 91 (1.55)/170 (1.43) | 1.19 (0.86-1.64) | 0.59 |
|  |  | No | 3780 (4.38)/8056 (4.94) | 1.01 (0.97-1.06) |  | 1045 (1.19)/2207 (1.32) | 1.08 (0.99-1.18) |  |
|  | Hematological malignancy | Yes | 20 (0.35)/32 (0.27) | 1.70 (0.79-3.65) | 0.25 | 6 (0.10)/12 (0.10) | 5.36 (0.35-82) | 0.31 |
|  |  | No | 265 (0.31)/547 (0.34) | 1.08 (0.91-1.28) |  | 75 (0.09)/150 (0.09) | 1.29 (0.92-1.81) |  |
|  | Hormone-related cancer | Yes | 85 (1.48)/186 (1.60) | 1.16 (0.84-1.59) | 0.30 | 10 (0.17)/30 (0.25) | 0.94 (0.33-2.70) | 0.86 |
|  |  | No | 1311 (1.52)/2958 (1.82) | 0.97 (0.90-1.05) |  | 164 (0.19)/313 (0.19) | 1.03 (0.81-1.32) |  |
|  | Immune-related cancer | Yes | 51 (0.89)/90 (0.77) | 1.14 (0.73-1.79) | 0.46 | 13 (0.22)/26 (0.22) | 2.28 (0.60-8.69) | 0.32 |
|  |  | No | 640 (0.74)/1347 (0.83) | 0.96 (0.86-1.07) |  | 147 (0.17)/265 (0.16) | 1.16 (0.90-1.49) |  |
|  | Smoking or alcohol-related cancer | Yes | 79 (1.37)/129 (1.11) | 1.39 (0.96-2.00) | 0.26 | 40 (0.68)/85 (0.71) | 0.75 (0.44-1.28) | 0.15 |
|  |  | No | 888 (1.03)/1757 (1.08) | 1.12 (1.01-1.24) |  | 501 (0.57)/1012 (0.61) | 1.12 (0.98-1.28) |  |

^a^ Per 1000 person years.

^b^ P value for subgroup difference was derived from the Wald test.

^c^ Cox regression models were stratified by matching identifiers (birth year and sex) and adjusted for educational level, family income level, marital status, family history of specific cancer type, Charlson comorbidity index, and history of substance use disorders.

^d^ Cox regression models were stratified by family identifiers and adjusted for birth year, sex, educational level, family income level, marital status, Charlson comorbidity index, and history of substance use disorders.

**Supplementary Table 7. Hazard ratios (HRs) and 95% confidence intervals (CIs)^a^ for cancer incidence and mortality among patients with stress-related disorders, compared to their matched unexposed individuals, additionally adjusted for the number of health care visits during the first year after cohort entry**

| **Cancer types** | **Cancer incidence** | | **Cancer mortality** | |
| --- | --- | --- | --- | --- |
|  | **No of cases (incidence^b^) in exposed/unexposed individuals** | **HR (95% CI)** | **No of cases (mortality^b^) in exposed/unexposed individuals** | **HR (95% CI)** |
| Any cancer | 6918 (4.64)/65742 (4.41) | 1.01 (0.99-1.03) | 2006 (1.32)/15756 (1.04) | 1.09 (1.04-1.15) |
| Hematological malignancy | 461 (0.31)/4522 (0.30) | 1.00 (0.90-1.10) | 126 (0.08)/1153 (0.08) | 0.90 (0.73-1.10) |
| Hormone-related cancer | 2418 (1.62)/25722 (1.73) | 0.99 (0.94-1.03) | 316 (0.21)/2625 (0.17) | 1.15 (1.01-1.30) |
| Immune-related cancer | 1163 (0.78)/12239 (0.82) | 0.91 (0.85-0.97) | 253 (0.17)/2055 (0.14) | 0.93 (0.80-1.07) |
| Smoking and alcohol-related cancer | 1658 (1.11)/11506 (0.77) | 1.15 (1.09-1.22) | 964 (0.63)/6422 (0.42) | 1.18 (1.10-1.27) |

^a^ Cox regression models were stratified by matching identifiers (birth year and sex) and adjusted for educational level, family income level, marital status, family history of specific cancer type, Charlson comorbidity index, history of substance use disorders, and the number of health care visits during first year after cohort entry (as continuous variable).

^b^ Per 1000 person years.

**Supplementary Table 8. Hazard ratios (HRs) and 95% confidence intervals (CIs)^a^ for cancer incidence and mortality among patients with stress-related disorders, compared to their matched unexposed individuals, using different lag-time and by different types of cancer**

| **Lag time** | **Cancer types** | **Cancer incidence** | | **Cancer mortality** | |
| --- | --- | --- | --- | --- | --- |
|  |  | **No of cases (incidence^b^) in exposed/unexposed individuals** | **HR (95% CI)** | **No of cases (mortality^b^) in exposed/unexposed individuals** | **HR (95% CI)** |
| 2 years after cohort entry was excluded | Any cancer | 6419 (4.83)/60559 (4.59) | 1.03 (1.00-1.05) | 1882 (1.39)/14479 (1.07) | 1.13 (1.07-1.19) |
|  | Hematological malignancy | 430 (0.32)/4122 (0.31) | 1.05 (0.95-1.17) | 116 (0.09)/1046 (0.08) | 1.07 (0.87-1.30) |
|  | Hormone-related cancer | 2239 (1.68)/23834 (1.81) | 0.98 (0.94-1.03) | 297 (0.22)/2396 (0.18) | 1.22 (1.07-1.38) |
|  | Immune-related cancer | 1062 (0.80)/11214 (0.85) | 0.92 (0.87-0.99) | 227 (0.17)/1886 (0.14) | 0.98 (0.84-1.13) |
|  | Smoking and alcohol-related cancer | 1563 (1.18)/10620 (0.80) | 1.18 (1.12-1.25) | 912 (0.67)/5926 (0.44) | 1.21 (1.12-1.30) |
| 5 years after cohort entry was excluded | Any cancer | 5134 (5.53)/47139 (5.20) | 1.03 (1.00-1.06) | 1526 (1.61)/11288 (1.22) | 1.15 (1.08-1.21) |
|  | Hematological malignancy | 330 (0.36)/3185 (0.35) | 1.02 (0.90-1.14) | 93 (0.10)/813 (0.09) | 1.07 (0.85-1.34) |
|  | Hormone-related cancer | 1789 (1.93)/18637 (2.06) | 0.98 (0.93-1.03) | 230 (0.24)/1812 (0.20) | 1.21 (1.04-1.39) |
|  | Immune-related cancer | 812 (0.87)/8561 (0.94) | 0.90 (0.84-0.97) | 180 (0.19)/1452 (0.16) | 1.00 (0.84-1.18) |
|  | Smoking and alcohol-related cancer | 1294 (1.39)/8420 (0.93) | 1.21 (1.14-1.29) | 748 (0.79)/4706 (0.51) | 1.21 (1.11-1.32) |
| 10 years after cohort entry was excluded | Any cancer | 3288 (6.88)/28735 (6.31) | 1.04 (1.00-1.08) | 1037 (2.12)/7017 (1.50) | 1.21 (1.13-1.30) |
|  | Hematological malignancy | 201 (0.42)/1868 (0.41) | 1.00 (0.86-1.16) | 57 (0.12)/497 (0.11) | 1.04 (0.78-1.40) |
|  | Hormone-related cancer | 1120 (2.35)/11538 (2.53) | 0.96 (0.90-1.02) | 143 (0.29)/1077 (0.23) | 1.26 (1.04-1.51) |
|  | Immune-related cancer | 509 (1.07)/4998 (1.10) | 0.90 (0.83-1.01) | 124 (0.25)/876 (0.19) | 1.14 (0.92-1.40) |
|  | Smoking and alcohol-related cancer | 907 (1.90)/5403 (1.19) | 1.28 (1.18-1.38) | 539 (1.10)/3050 (0.65) | 1.32 (1.19-1.45) |

^a^ Cox regression models were stratified by matching identifiers (birth year and sex) and adjusted for educational level, family income level, marital status, family history of specific cancer type, Charlson comorbidity index, and history of substance use disorders.

^b^ Per 1000 person years.

**Supplementary Table 9. Hazard ratios (HRs) and 95% confidence intervals (CIs) for cancer incidence and mortality among patients with acute stress reaction, compared to their matched unexposed individuals and unaffected full siblings, additionally adjusted for the number of health care visits during the first year after cohort entry or using different lag-time**

|  | **Model information** | **Cancer incidence** | | **Cancer mortality** | |
| --- | --- | --- | --- | --- | --- |
|  |  | **No of cases (incidence^a^) in exposed/unexposed individuals** | **HR (95% CI)** | **No of cases (mortality^a^) in exposed/unexposed individuals** | **HR (95% CI)** |
| **Population-matched cohort^b^** | Additionally adjusted for the number of health care visits during the first year after cohort entry | 2836 (4.53)/26869 (4.30) | 0.99 (0.95-1.04) | - | - |
|  | 2 years after cohort entry was excluded | 2621 (4.71)/24680 (4.48) | 1.02 (0.97-1.06) | 771 (1.36)/5744 (1.02) | 1.13 (1.04-1.23) |
|  | 5 years after cohort entry was excluded | 2083 (5.46)/18908 (5.10) | 1.03 (0.98-1.08) | 617 (1.58)/4360 (1.15) | 1.15 (1.05-1.26) |
|  | 10 years after cohort entry was excluded | 1316 (6.95)/11192 (6.27) | 1.05 (0.98-1.11) | 391 (2.02)/2552 (1.40) | 1.18 (1.05-1.32) |
| **Sibling cohort^c^** | Additionally adjusted for the number of health care visits during the first year after cohort entry | 1699 (4.40)/3599 (4.91) | 1.02 (0.95-1.09) | - | - |
|  | 2 years after cohort entry was excluded | 1573 (4.60)/3329 (5.11) | 1.04 (0.97-1.11) | 451 (1.30)/915 (1.37) | 1.16 (1.01-1.33) |
|  | 5 years after cohort entry was excluded | 1223 (5.24)/2578 (5.73) | 1.05 (0.97-1.14) | 344 (1.45)/715 (1.55) | 1.11 (0.94-1.30) |
|  | 10 years after cohort entry was excluded | 757 (6.57)/1579 (6.96) | 1.06 (0.96-1.17) | 218 (1.85)/411 (1.77) | 1.16 (0.94-1.42) |

^a^ Per 1000 person years.

^b^ Cox regression models were stratified by matching identifiers (birth year and sex) and adjusted for educational level, family income level, marital status, family history of cancer, Charlson comorbidity index, and history of substance use disorders.

^c^ Cox regression models were stratified by family identifiers and adjusted for birth year, sex, educational level, family income level, marital status, Charlson comorbidity index, and history of substance use disorders.

**Supplementary Table 10. Subhazard ratios (SHRs) and 95% confidence intervals (CIs) for cancer mortality among patients with stress-related disorders, compared to their matched unexposed individuals and unaffected full siblings**

|  | **Cancer types** | **Cancer mortality** | |
| --- | --- | --- | --- |
|  |  | **No of cases (mortality^a^) in exposed/unexposed individuals** | **SHR^b^ (95% CI)** |
| **Population-matched cohort** | Any cancer | 2006 (1.39)/15756 (1.09) | 1.06 (1.01-1.11) |
|  | Hematological malignancy | 126 (0.09)/1153 (0.08) | 1.00 (0.84-1.20) |
|  | Hormone-related cancer | 316 (0.22)/2625 (0.18) | 1.12 (1.00-1.26) |
|  | Immune-related cancer | 253 (0.17)/2055 (0.14) | 0.98 (0.86-1.12) |
|  | Smoking and alcohol-related cancer | 964 (0.67)/6422 (0.44) | 1.12 (1.05-1.20) |
| **Sibling cohort** | Any cancer | 1136 (1.27)/2377 (1.41) | 1.07 (1.00-1.14) |
|  | Hematological malignancy | 81 (0.09)/162 (0.10) | 1.23 (0.95-1.59) |
|  | Hormone-related cancer | 174 (0.19)/343 (0.20) | 1.05 (0.88-1.26) |
|  | Immune-related cancer | 160 (0.18)/291 (0.17) | 1.14 (0.95-1.38) |
|  | Smoking and alcohol-related cancer | 541 (0.60)/1097 (0.65) | 1.08 (0.97-1.19) |

^a^ Per 1000 person years.

^b^ In the population-matched cohort, SHRs were derived from modified Cox models, stratified by matching identifiers (birth year and sex) and adjusted for educational level, family income level, marital status, family history of specific cancer type, Charlson comorbidity index, and history of substance use disorders. In the sibling cohort, SHRs were derived from modified Cox models, stratified by family identifiers and adjusted for birth year, sex, educational level, family income level, marital status, Charlson comorbidity index, and history of substance use disorders.
